# Author Correction: Targeting HDAC6 to treat heart failure with preserved ejection fraction in mice

**DOI:** 10.1038/s41467-024-52831-3

**Published:** 2024-10-09

**Authors:** Sara Ranjbarvaziri, Aliya Zeng, Iris Wu, Amara Greer-Short, Farshad Farshidfar, Ana Budan, Emma Xu, Reva Shenwai, Matthew Kozubov, Cindy Li, Melissa Van Pell, Francis Grafton, Charles E MacKay, Xiaomei Song, James R Priest, Gretchen Argast, Mohammad A. Mandegar, Timothy Hoey, Jin Yang

**Affiliations:** Tenaya Therapeutics, South San Francisco, CA USA

**Keywords:** Target identification, Heart failure, Target validation, Heart failure, Mechanisms of disease

Correction to: *Nature Communications* 10.1038/s41467-024-45440-7, published online 26 February 2024

The original version of this Article contained errors in Fig. 2 panel d, which occurred during final preparation of the figures and don’t affect the conclusions. In Figure 2d mistakenly included Left Ventricle samples in the healthy control group, when only Right Ventricle samples should have been included for comparison with the HFpEF right ventricle samples. The authors reanalyzed the data, including only right ventricle samples for the controls and right ventricle HFpEF samples.

The correct version of Fig. 2d is:
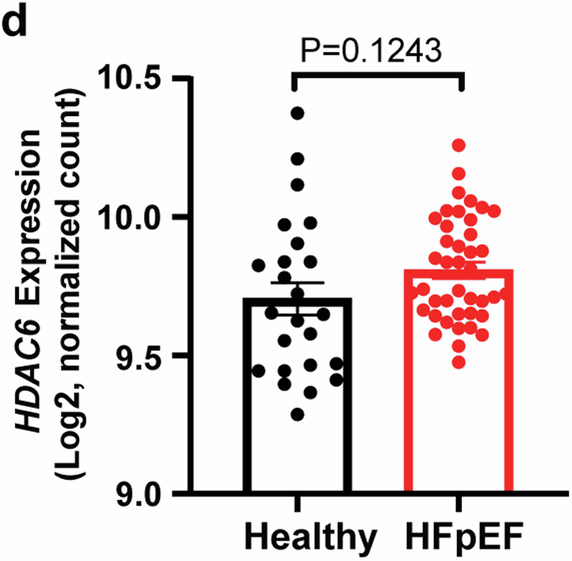


Which replaces the incorrect version of Fig. 2d:
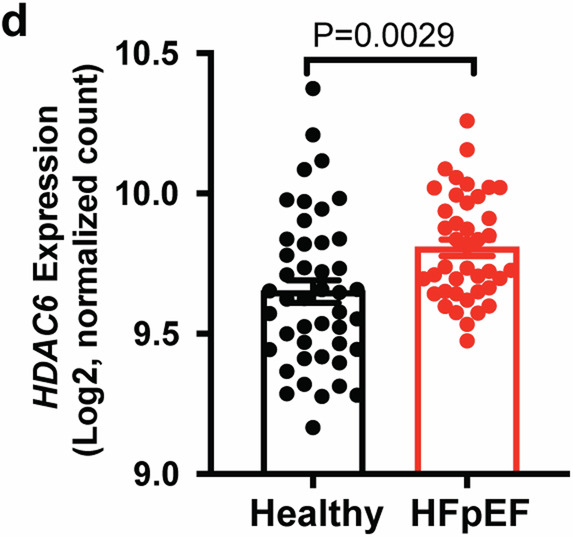


The main text included this sentence: ‘Consistent with HFD+mTAC and HFD + L-NAME mice, HDAC6 expression was significantly higher in human hearts with HFpEF versus controls (Fig. 2d). These findings support that changes in HDAC6 expression in HFpEF are evolutionarily conserved.’ The correct version replaces this with ‘ HDAC6 mRNA levels were trending increased (+7.4%) in human hearts with HFpEF versus controls, although this difference is not statistically significant (Fig. 2d). Future studies should incorporate more data points to achieve a robust statistical comparison. Furthermore, given protein levels can provide additional insights into the functional status of HDAC6 beyond mRNA expression, it is crucial to analyze HDAC6 protein levels in HFpEF patient hearts, as have done in the mouse hearts of the two HFpEF models (Fig. 2b and 2c).’

The figure legend included the following text: ‘d Quantitation of HDAC6 mRNA expression in human heart tissues of healthy (n = 45) and patients with HFpEF (n = 41) from published RNA-Seq data. Data are expressed as the mean ± SEM. Statistical significance was assessed by unpaired two-sided Student’s t test (b, d, e, g–j). ‘. The correct version replaces this with ‘Quantitation of HDAC6 mRNA expression in human heart tissues of healthy (n = 24) and patients with HFpEF (n = 41) from published RNA-Seq data. Data are expressed as the mean ± SEM. Statistical significance was assessed by unpaired two-sided Student’s t test (b, c), or unpaired two-sided t test with Welch’s correction (d)’

The Source Data File has been updated accordingly.

This has been corrected in both the PDF and HTML versions of the Article.

## Source data

Revised Source Data

