# Supplementary material for: Targeting HDAC6 to treat heart failure with preserved ejection fraction in mice
Source: Nat Commun. 2024 Feb 26;15:1352. doi: 10.1038/s41467-024-45440-7 (PMC10897156; doi:10.1038/s41467-024-45440-7)
Supplement: Supplementary file 1 — Supplementary Information [file 41467_2024_45440_MOESM1_ESM.pdf]

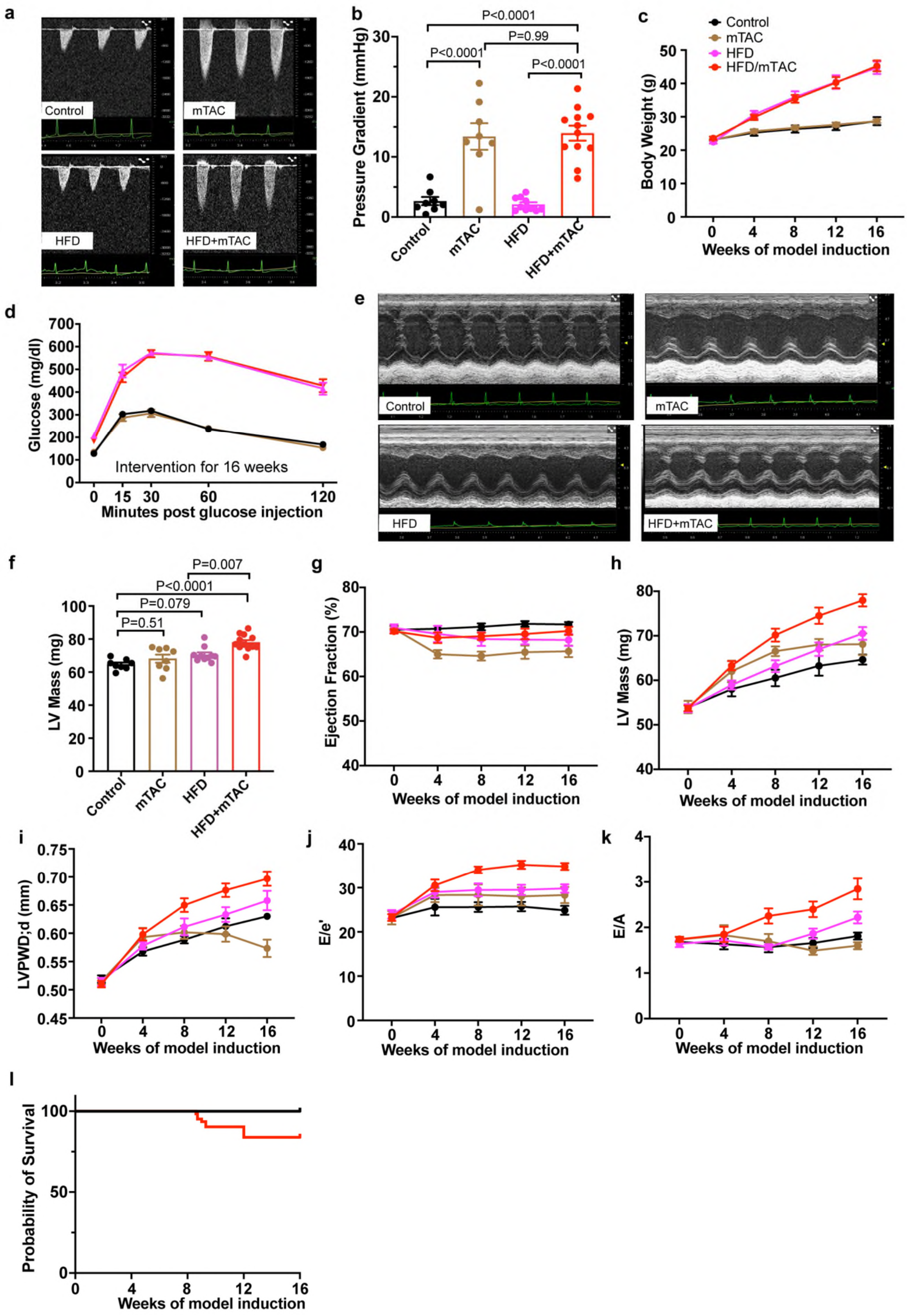

a
Control
mTAC
HFD
HFD+mTAC
b
Pressure Gradient (mmHg)
P<0.0001
P<0.0001
P=0.99
P<0.0001
Control
mTAC
HFD
HFD+mTAC
c
Control
mTAC
HFD
HFD/mTAC
Body Weight (g)
Weeks of model induction
d
Glucose (mg/dl)
Intervention for 16 weeks
Minutes post glucose injection
e
Control
mTAC
HFD
HFD+mTAC
f
LV Mass (mg)
P=0.007
P<0.0001
P=0.079
P=0.51
Control
mTAC
HFD
HFD+mTAC
g
Ejection Fraction (%)
Weeks of model induction
h
LV Mass (mg)
Weeks of model induction
i
LVPWD;d (mm)
Weeks of model induction
j
E/e'
Weeks of model induction
k
E/A
Weeks of model induction
l
Probability of Survival
Weeks of model induction

Supplementary Fig. 1. Characterization of the HFD+mTAC model.
a, Representative pulsed-wave Doppler images of the aortic arch. **b**, Quantitation of pressure gradient in the experimental groups (control *n*=8, mTAC *n*=8, HFD *n*=10, HFD+mTAC *n*=12 mice). **c**, Body weight of mice in each group recorded every 4 weeks (control *n*=8, mTAC *n*=8, HFD *n*=10, HFD+mTAC *n*=12 mice). **d**, Intraperitoneal glucose-tolerance test 16 weeks after induction (control *n*=12, mTAC *n*=8, HFD *n*=10, HFD+mTAC *n*=10 mice). **e**, Representative Echocardiography M-Mode images. **f**, Echocardiographic measurement of LV mass 16 weeks after induction (control *n*=8, mTAC *n*=8, HFD *n*=10, HFD+mTAC *n*=12 mice). **g**-**k**, Longitudinal follow up of (**g**) EF, (**h**) LV mass, (**i**) LVPWd, (**j**) E/e', and (**k**) E/A with echocardiography every 4 weeks until 16 weeks after induction (control *n*=8, mTAC *n*=8, HFD *n*=10, HFD+mTAC *n*=12 mice). **l**, Kaplin Meier curve of control and HFD+mTAC mice. (control *n*=12, mTAC *n*=8, HFD *n*=8, HFD+mTAC *n*=62 mice). Data are expressed as the mean ± SEM. Statistical analysis was performed using one-way ANOVA followed by Tukey's multiple comparisons test (**b**, **f**). The exact *P* values are shown in the figures. NS, not significant. Source data are provided as a Source Data file.

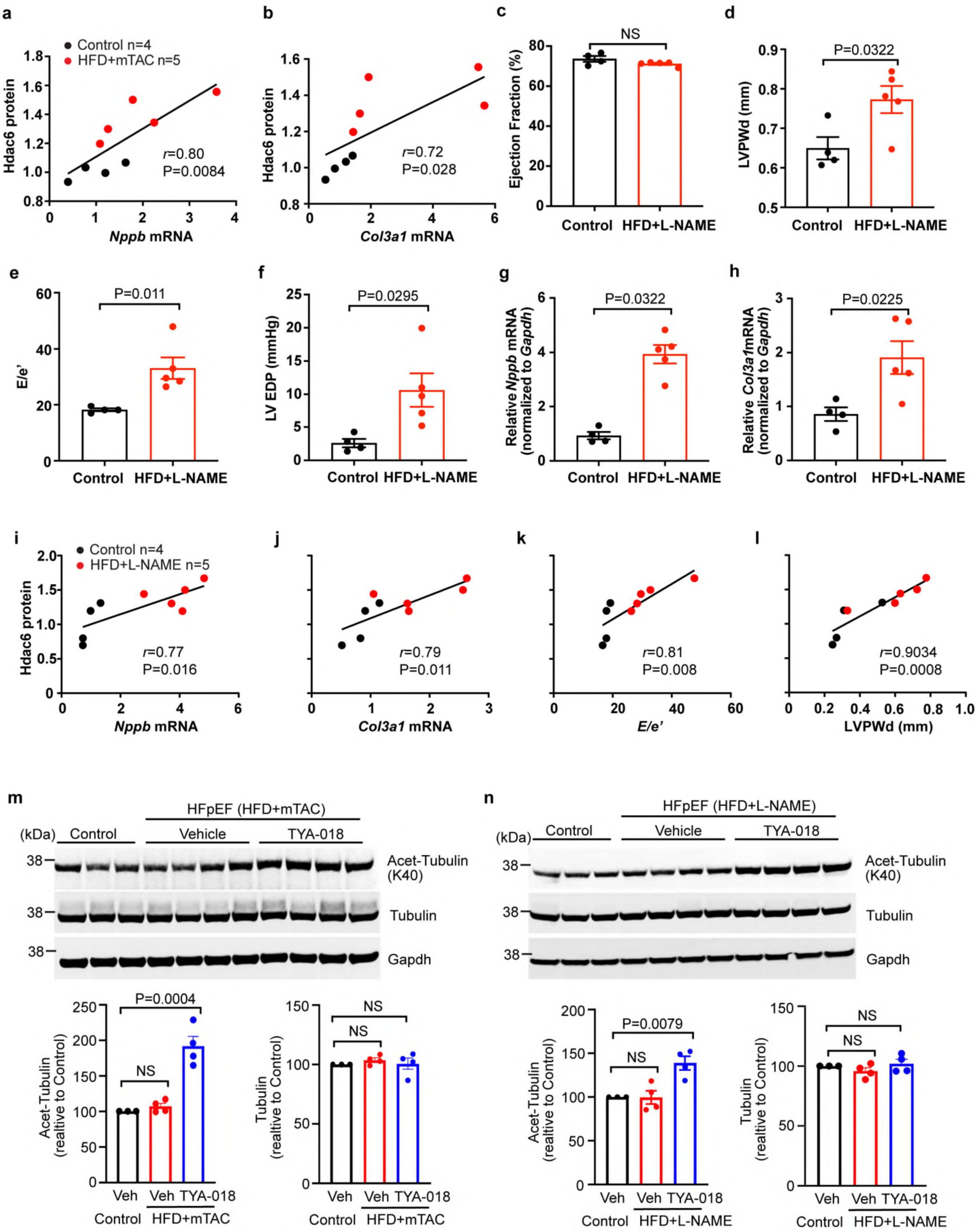
a
Control n=4
HFD+mTAC n=5
Hdac6 protein
r=0.80
P=0.0084
Nppb mRNA
b
Hdac6 protein
r=0.72
P=0.028
Col3a1 mRNA
c
NS
Ejection Fraction (%)
Control
HFD+L-NAME
d
P=0.0322
LVPWd (mm)
Control
HFD+L-NAME
e
P=0.011
E/e'
Control
HFD+L-NAME
f
P=0.0295
LV EDP (mmHg)
Control
HFD+L-NAME
g
P=0.0322
Relative Nppb mRNA (normalized to Gapdh)
Control
HFD+L-NAME
h
P=0.0225
Relative Col3a1mRNA (normalized to Gapdh)
Control
HFD+L-NAME
i
Control n=4
HFD+L-NAME n=5
Hdac6 protein
r=0.77
P=0.016
Nppb mRNA
j
r=0.79
P=0.011
Col3a1 mRNA
k
r=0.81
P=0.008
E/e'
l
r=0.9034
P=0.0008
LVPWd (mm)
m
HFpEF (HFD+mTAC)
(kDa)
Control
Vehicle
TYA-018
Acet-Tubulin (K40)
Tubulin
Gapdh
P=0.0004
NS
Acet-Tubulin (realtive to Control)
Veh
Veh TYA-018
Control
HFD+mTAC
NS
NS
Tubulin (realtive to Control)
Veh
Veh TYA-018
Control
HFD+mTAC
n
HFpEF (HFD+L-NAME)
(kDa)
Control
Vehicle
TYA-018
Acet-Tubulin (K40)
Tubulin
Gapdh
P=0.0079
NS
Acet-Tubulin (realtive to Control)
Veh
Veh TYA-018
Control
HFD+L-NAME
NS
NS
Tubulin (realtive to Control)
Veh
Veh TYA-018
Control
HFD+L-NAME

Supplementary Fig. 2. HDAC6 is upregulated in HFpEF hearts and correlates with heart failure marker genes and echocardiography parameters.
Correlation of Hdac6 protein with (**a**) *Nppb* and (**b**) *Col3a1* mRNA expression in hearts of control (*n*=4) and HFD+mTAC (*n*=5) mice. **c**-**e**, Echocardiographic measurement of (**c**) EF, (**d**) LVPWd, and (**e**) E/e' in HFD+L-NAME mice. (**f**) EDP measured using an in vivo catheter in control (*n*=4) and HFD+L-NAME (*n*=5) mice. Quantitation of (**g**) *Nppb* and (**h**) *Col3a1* mRNA expression in control (*n*=4) and HFD+L-NAME (*n*=5) mice. Correlation of Hdac6 protein with (**i**) *Nppb* and (**j**) *Col3a1* mRNA expression in heart tissues of control (*n*=4) and HFD+L-NAME (*n*=5) mice. Correlation of HDAC6 protein with (**k**) E/e' and (**l**) LVPWd in control (*n*=4) and HFD+L-NAME (*n*=5) mice. Western blot analysis of Acetyl-tubulin, Tubulin, Gapdh in mouse hearts of control (n=3), HFD+mTAC (**m**) and HFD+L-NAME (**n**) model treated with vehicle (n=4) or TYA-018 15mg/kg (n=4). Data are expressed as the mean ± SEM. The two-tailed *P* value for Pearson correlation coefficient (*r*) (**a**, **b**, **i-l**). Statistical significance was assessed by unpaired two-sided Student's t test (**c**-**h**) or one-way ANOVA followed by Tukey's multiple comparisons test (**m**, **n**). The exact *P* and *r* values are shown in the figures. NS, not significant. Source data are provided as a Source Data file.

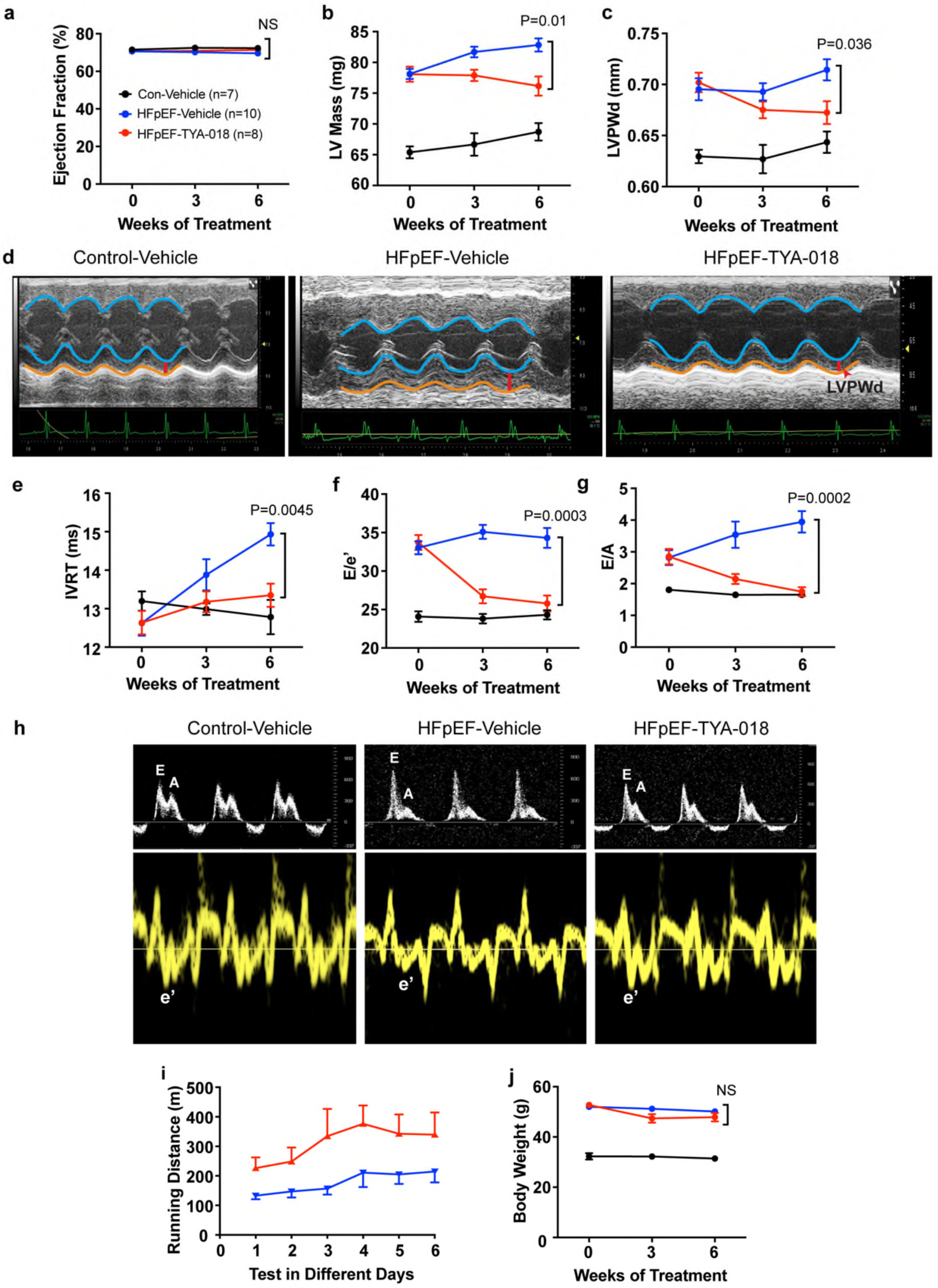

Supplementary Fig. 3. Time course of improvements in heart function in HFpEF model after TYA-018 treatment.

a-g, Longitudinal follow up of EF, LV mass, LVPWd, IVRT, E/e’, and E/A using echocardiography 3 weeks and 6 weeks after treatment (Control *n*=7, HFD+mTAC-

Vehicle *n*=10, HFD+mTAC-TYA-018 *n*=8 mice). Representative LV M-mode echocardiographic tracings after 6 weeks of treatment (**d**). Images are representative of 7–10 mice. **h**, Representative pulsed-wave Doppler (top) and tissue Doppler (bottom) tracings after 6 weeks of treatment. Images are representative of 7–10 mice. **i**, Running distance recording of exercise exhaustion test over six days. **j**, Body weight of mice recorded every 3 weeks. Data are expressed as the mean ± SEM. Statistical analysis was performed using two-way ANOVA followed by Tukey's multiple comparisons test (**a-c**, **e-g, o**). The exact *P* values of comparison between group HFpEF-Veh vs. HFpEF-TYA-018 are shown in the figures. NS, not significant. Source data are provided as a Source Data file.

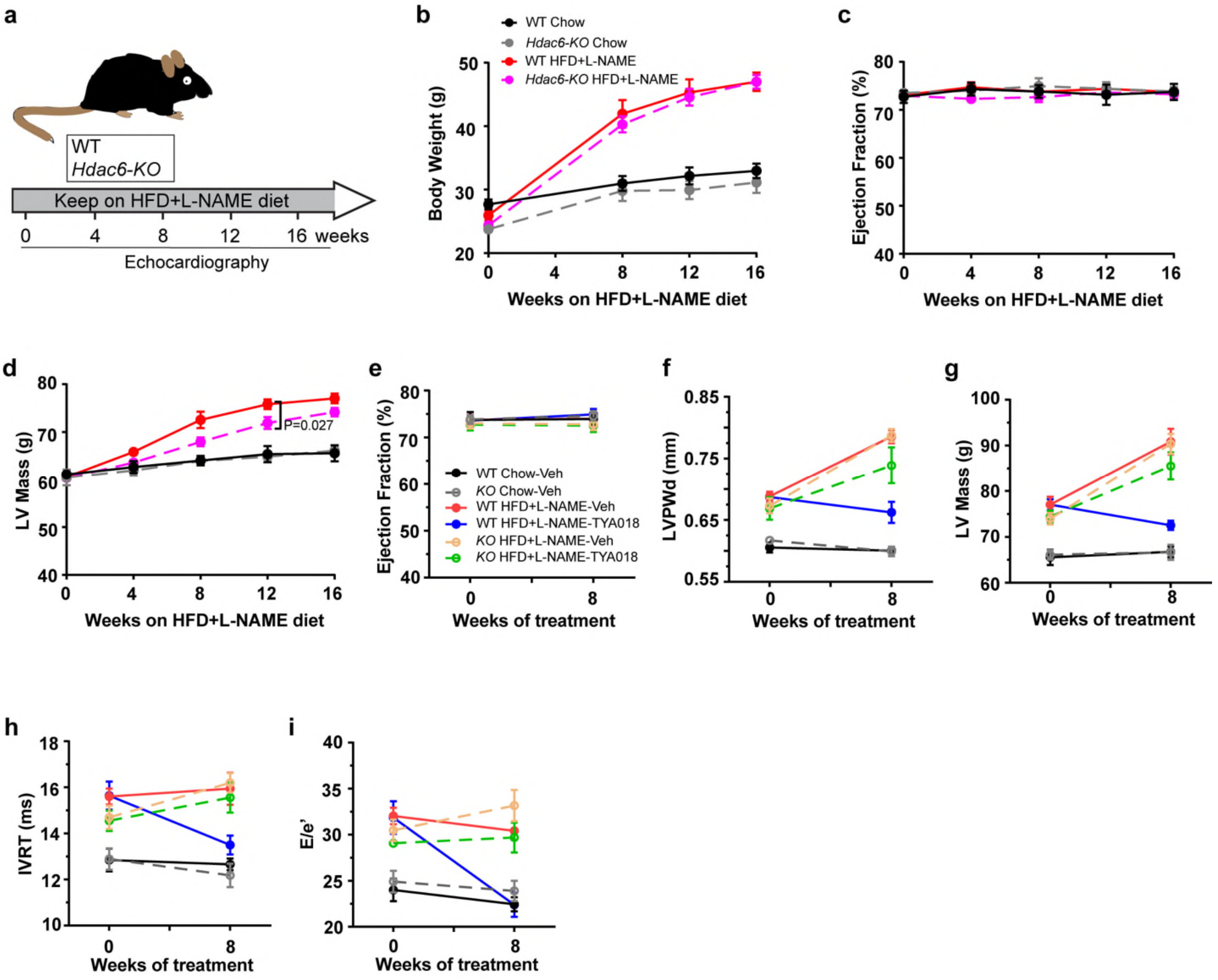

Supplementary Fig. 4. *Hdac6-KO* mice develop less severe HFpEF phenotypes, and not response to TYA-018 treatment.
a, Schematic overview of study design. *Hdac6* knockout (KO) and wild-type littermates treated with HFD+L-NAME for 16 weeks. Echocardiography was performed every 4 weeks after treatment. **b**-**f**, Time course of (**b**) body weight and echocardiography measurements of (**c**) EF, (**d**) LV mass by 16 weeks of HFD+L-NAME treatment. **E**-**I**, Time course of echocardiography measurements of (**e**) EF, (**f**) LVPWd, (**g**) LV mass, (**h**) IVRT and (**i**) E/e' by 8 weeks of TYA-018 treatment. Data are expressed as the mean ± SEM. Statistical analysis was performed using two-way ANOVA followed by Tukey's multiple comparisons test (**d**). The exact *P* values of comparison between group WT-HFD+L-NAME vs. KO-HFD+L-NAME are shown in the figures. NS, not significant. Source data are provided as a Source Data file.

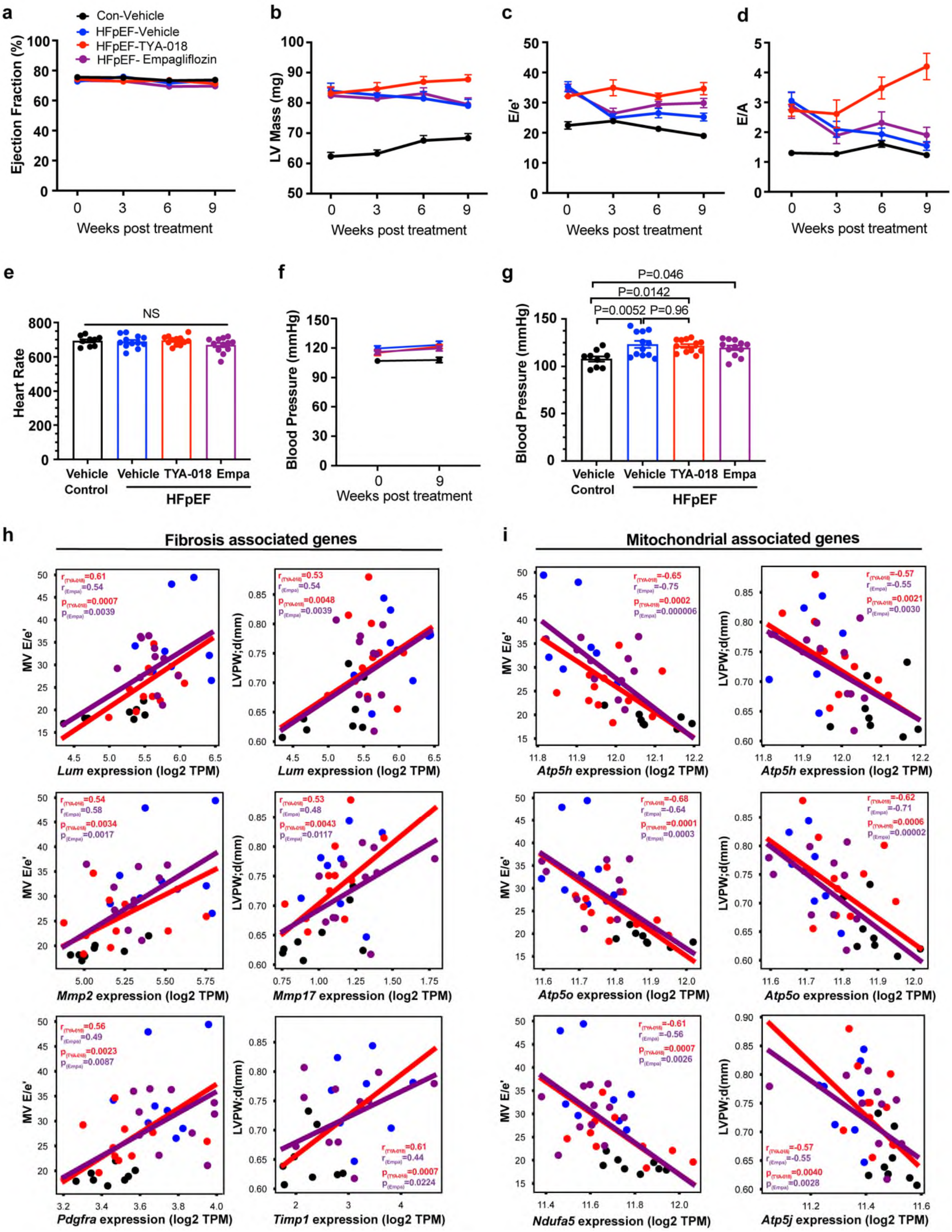
a
Con-Vehicle
HFpEF-Vehicle
HFpEF-TYA-018
HFpEF- Empagliflozin
Ejection Fraction (%)
Weeks post treatment
b
LV Mass (mg)
Weeks post treatment
c
E/e'
Weeks post treatment
d
E/A
Weeks post treatment
e
NS
Heart Rate
Vehicle
Control
Vehicle TYA-018 Empa
HFpEF
f
Blood Pressure (mmHg)
Weeks post treatment
g
P=0.046
P=0.0142
P=0.0052 P=0.96
Blood Pressure (mmHg)
Vehicle
Control
Vehicle TYA-018 Empa
HFpEF
h
Fibrosis associated genes
r(TYA-018)=0.61
r(Empa)=0.54
p(TYA-018)=0.0007
p(Empa)=0.0039
MV E/e'
Lum expression (log2 TPM)
r(TYA-018)=0.53
r(Empa)=0.54
p(TYA-018)=0.0048
p(Empa)=0.0039
LVPW;d(mm)
Lum expression (log2 TPM)
r(TYA-018)=0.54
r(Empa)=0.58
p(TYA-018)=0.0034
p(Empa)=0.0017
MV E/e'
Mmp2 expression (log2 TPM)
r(TYA-018)=0.53
r(Empa)=0.48
p(TYA-018)=0.0043
p(Empa)=0.0117
LVPW;d(mm)
Mmp17 expression (log2 TPM)
r(TYA-018)=0.56
r(Empa)=0.49
p(TYA-018)=0.0023
p(Empa)=0.0087
MV E/e'
Pdgfra expression (log2 TPM)
r(TYA-018)=0.61
r(Empa)=0.44
p(TYA-018)=0.0007
p(Empa)=0.0224
LVPW;d(mm)
Timp1 expression (log2 TPM)
i
Mitochondrial associated genes
r(TYA-018)=-0.65
r(Empa)=-0.75
p(TYA-018)=0.0002
p(Empa)=0.000006
MV E/e'
Atp5h expression (log2 TPM)
r(TYA-018)=-0.57
r(Empa)=-0.55
p(TYA-018)=0.0021
p(Empa)=0.0030
LVPW;d(mm)
Atp5h expression (log2 TPM)
r(TYA-018)=-0.68
r(Empa)=-0.64
p(TYA-018)=0.0001
p(Empa)=0.0003
MV E/e'
Atp5o expression (log2 TPM)
r(TYA-018)=-0.62
r(Empa)=-0.71
p(TYA-018)=0.0006
p(Empa)=0.00002
LVPW;d(mm)
Atp5o expression (log2 TPM)
r(TYA-018)=-0.61
r(Empa)=-0.56
p(TYA-018)=0.0007
p(Empa)=0.0026
MV E/e'
Ndufa5 expression (log2 TPM)
r(TYA-018)=-0.57
r(Empa)=-0.55
p(TYA-018)=0.0040
p(Empa)=0.0028
LVPW;d(mm)
Atp5j expression (log2 TPM)

Supplementary Fig. 5. TYA-018 demonstrated comparable efficacy to empagliflozin in HFD+L-NAME mice.
a-**d**, Longitudinal heart function measurements of (**a**) EF, (**b**) LV mass, (**c**) E/e' and (**d**) E/A with echocardiography after 3, 6, and 9 weeks of treatment with vehicle, TYA-018, or empagliflozin. **e**, heart rate after 9 weeks of treatment, **f**, time course of blood pressure at pre-dose and 9 weeks of treatment, **g**, blood pressure at 9 weeks of treatment. **h**, Correlation of E/e' and LVPWd with mRNA expression of the fibrosis-associated genes *Lum*, *Mmp2,* and *Pdgfra* in HFpEF mice treated with TYA-018 or empagliflozin. **i**, Correlation of E/e' and LVPWd with mRNA expression of the mitochondrial function–associated genes *Atp5h*, *Atp5o,* and *Ndufa5* in HFpEF mice treated with TYA-018 or empagliflozin. Data are expressed as the mean ± SEM. Statistical significance was assessed by unpaired one-way ANOVA followed by Tukey's multiple comparisons test (**e**, **g**). The two-tailed *P* value for Pearson correlation coefficient (*r*) (**h**, **i**). The exact *P* and *r* values are shown in the figures. Source data are provided as a Source Data file.

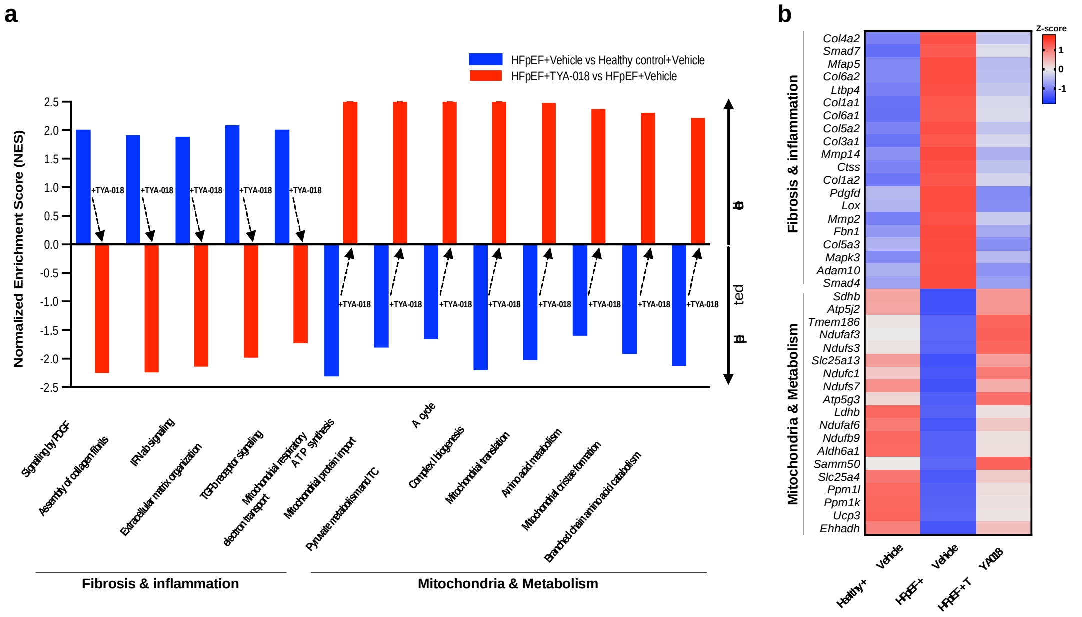

Supplementary Fig. 6. Single dose of TYA-018 corrects expression of gene sets associated with fibrosis and mitochondrial function.
a, GSEA of heart samples collected after one dose of vehicle or 15 mg/kg TYA-018 in HFD+L-NAME mice (*n* = 4 animals per group). Shown pathways were selected from MSigDB canonical pathways. **b**, Heatmap shows the expressional pattern of selected genes from gene set enrichment terms.

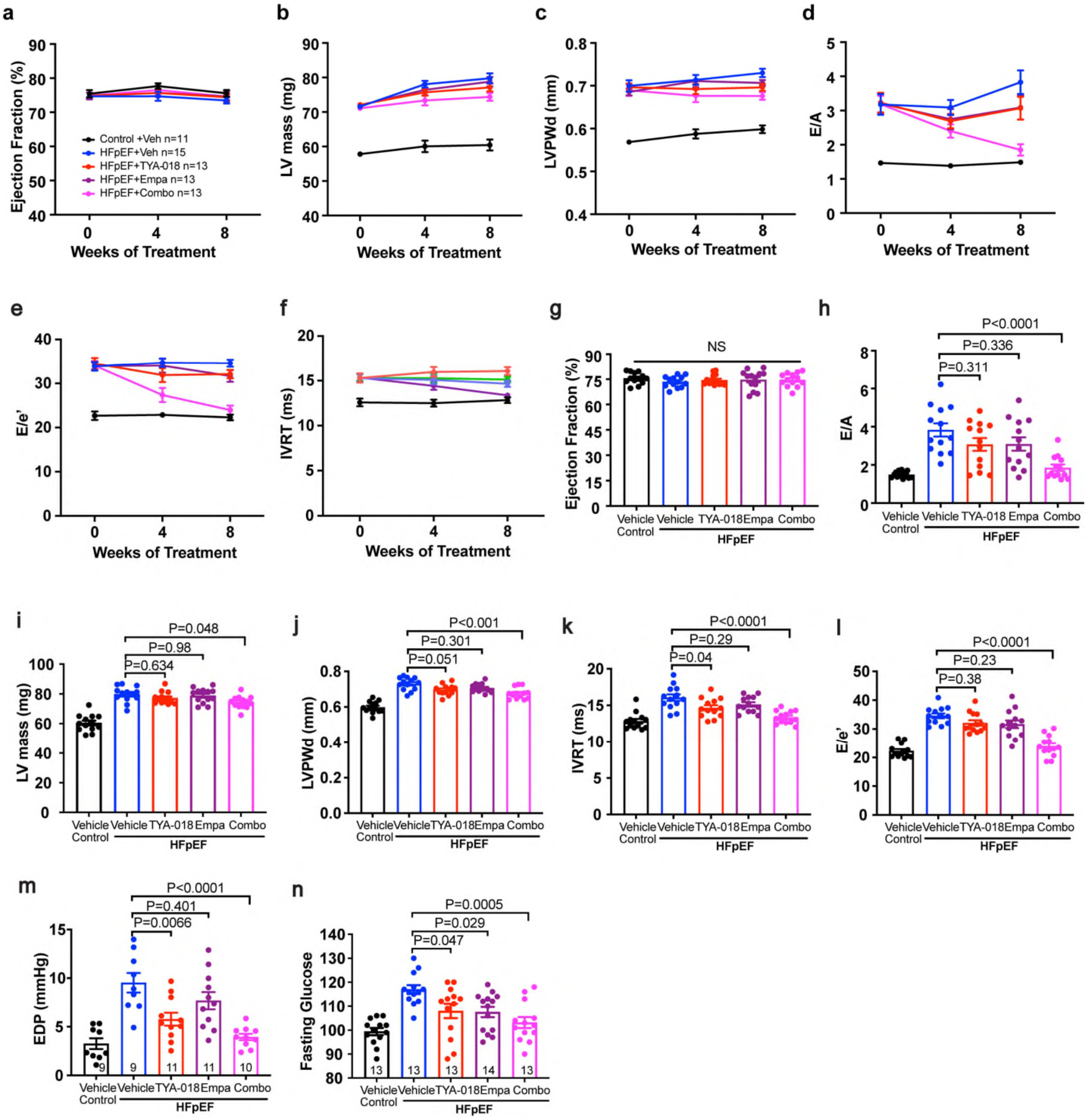

Supplementary Fig. 7. Combination Effects of TYA-018 and Empagliflozin in HFpEF

Longitudinal heart function measurements of (**a**) EF, (**b**) LV mass, (**c**) LVPWd, (**d**) E/A, (**e**) E/e' and (**f**) IVRT with echocardiography after 4, and 8 weeks of treatment with vehicle, 0.3mg/kg TYA-018, 0.5mg/kg empagliflozin or combination of TYA-018 and empagliflozin. Echocardiography quantitation of the (**g**) EF, (**h**) E/A, (**i**) LV Mass (**j**) LWPWd, (**k**) IVRT, (**l**) E/e', and (**m**) EDP after 8 weeks of treatment in combination therapy study in HFD+L-NAME HFpEF model. Mice number in each group is indicated

in panel **a**. (**n**) Quantitation of blood glucose at 6 hours after dosing with 0.3mg/kg TYA-018, 0.5mg/kg empagliflozin or combination of the two compounds and fasting. Mice number in each group is indicated in graphs (**m, n**). Data are expressed as the mean ± SEM. Statistical significance was assessed by one-way ANOVA followed by Tukey's multiple comparisons test (**h**-**n**). The exact *P* are shown in the figures. NS, not significant. Source data are provided as a Source Data file.

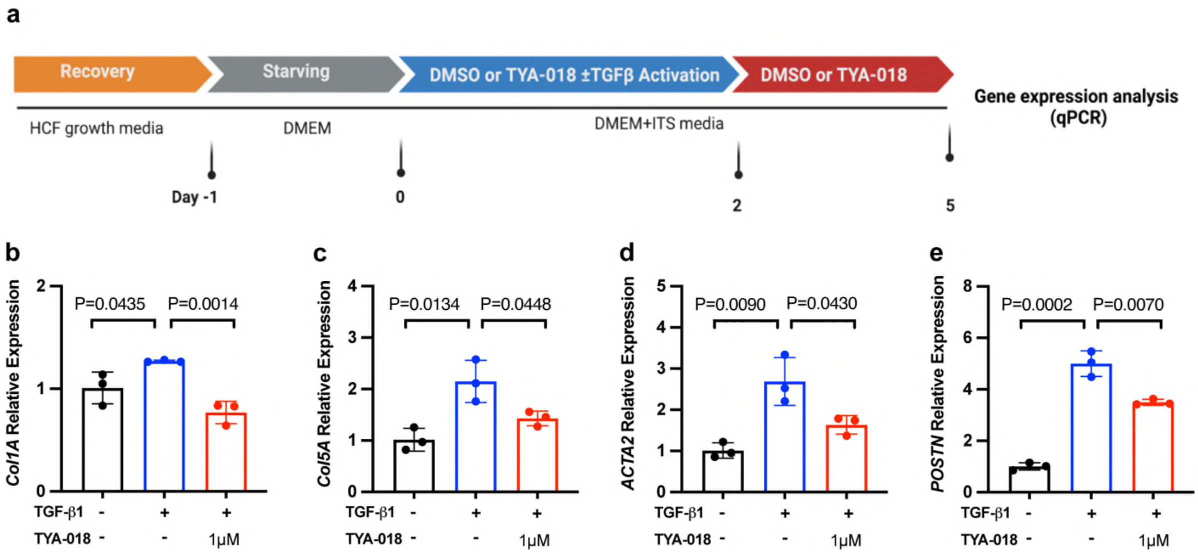

Supplementary Fig. 8. TYA-018 reduces activation of cardiac fibroblasts.
a, Schematic diagram of gene expression analysis of cardiac fibroblast activation assay in in vitro using TGF-β. **b**-**e**, qPCR analysis of *Col1a*, *Col5a*, *Acta2,* and *Postn* in cardiac fibroblasts treated with vehicle (n=3) or TYA-018 (1 µM) (n=3). Data are expressed as the mean ± SEM. Statistical significance was assessed by one-way ANOVA followed by Tukey's multiple comparisons test (**b**-**e**). The exact *P* values are shown in the figures. Source data are provided as a Source Data file.

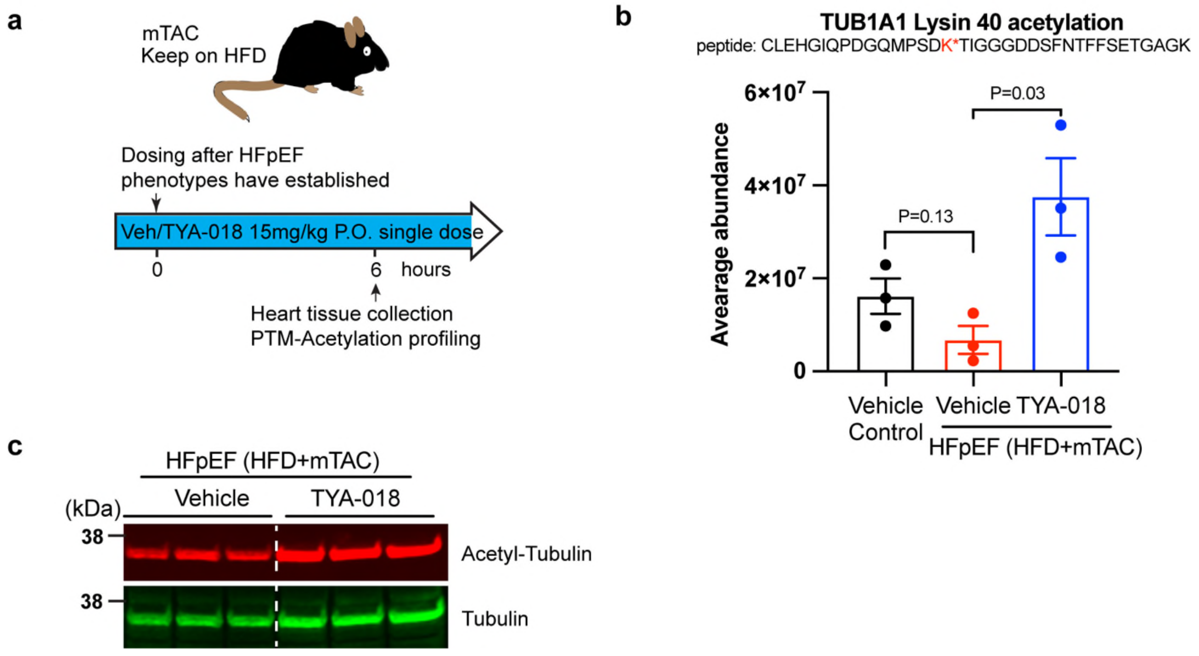

Supplementary Fig. 9. Potential substrates of HDAC6.
a, Schematic overview of heart sample preparation for PTM-acetylation profiling. **b**, Quantitation of TUB1A1 acetylation of heart tissues treated with vehicle (n=3) or 15 mg/kg TYA-018 (n=3). Data are expressed as the mean ± SEM. Statistical significance was assessed by one-way ANOVA followed by Tukey's multiple comparisons test. **c**, Western blot analysis of tubulin and acetyl-tubulin in heart samples from mice treated with vehicle or 15 mg/kg TYA-018. Source data are provided as a Source Data file.

Supplementary Table 1. TaqMan probes used for qPCR analysis

**Symbol**	**Gene name**	**TaqMan assay ID**
COL1A1	collagen type I alpha 1	Hs00164004_m1
COL5A	collagen type V alpha 1	Hs00609133_m1
POSTN	periostin	Hs01566734_m1
ACTA2	actin alpha 2, smooth muscle	Hs00426835_g1
GAPDH	glyceraldehyde 3-phosphate dehydrogenase	Hs02786624_g1
Nppb	Natriuretic peptide type B	Mm01255770_g1
Col3a1	collagen type III alpha 1	Mm00802300_m1
Gapdh	glyceraldehyde 3-phosphate dehydrogenase	Mm99999915_g1
