# Supplementary material for: Targeting HDAC6 to treat heart failure with preserved ejection fraction in mice
Source: Nat Commun. 2024 Feb 26;15:1352. doi: 10.1038/s41467-024-45440-7 (PMC10897156; doi:10.1038/s41467-024-45440-7)
Supplement: Supplementary file 2 — Reporting Summary [file 41467_2024_45440_MOESM2_ESM.pdf]

nature portfolio

Corresponding author(s): Jin Yang

Last updated by author(s): Nov 28, 2023

Reporting Summary

Nature Portfolio wishes to improve the reproducibility of the work that we publish. This form provides structure for consistency and transparency in reporting. For further information on Nature Portfolio policies, see our Editorial Policies and the Editorial Policy Checklist.

Statistics

For all statistical analyses, confirm that the following items are present in the figure legend, table legend, main text, or Methods section.

n/a	Confirmed	
☐	☒	The exact sample size (*n*) for each experimental group/condition, given as a discrete number and unit of measurement
☐	☒	A statement on whether measurements were taken from distinct samples or whether the same sample was measured repeatedly
☐	☒	The statistical test(s) used AND whether they are one- or two-sided<br>*Only common tests should be described solely by name; describe more complex techniques in the Methods section.*
☒	☐	A description of all covariates tested
☒	☐	A description of any assumptions or corrections, such as tests of normality and adjustment for multiple comparisons
☐	☒	A full description of the statistical parameters including central tendency (e.g. means) or other basic estimates (e.g. regression coefficient) AND variation (e.g. standard deviation) or associated estimates of uncertainty (e.g. confidence intervals)
☐	☒	For null hypothesis testing, the test statistic (e.g. *F*, *t*, *r*) with confidence intervals, effect sizes, degrees of freedom and *P* value noted<br>*Give P values as exact values whenever suitable.*
☒	☐	For Bayesian analysis, information on the choice of priors and Markov chain Monte Carlo settings
☒	☐	For hierarchical and complex designs, identification of the appropriate level for tests and full reporting of outcomes
☐	☒	Estimates of effect sizes (e.g. Cohen's *d*, Pearson's *r*), indicating how they were calculated

Our web collection on statistics for biologists contains articles on many of the points above.

Software and code

Policy information about availability of computer code

Data collection	Echocardiography image and data collection were performed using VisualSonics Vevo 2100 system. Western blot images were acquired by LI-COR odyssey system. Fluorescence images for TMRM, perchlorate assay were acquired iusing Cytation 5 (BioTek). RT-qPCR reactions were performed using the QuantStudio7 Flex Real-Time PCR systems (Thermo Fisher Scientific).
Data analysis	The statistical analysis was performed using GraphPad Prism (Version 10).

For manuscripts utilizing custom algorithms or software that are central to the research but not yet described in published literature, software must be made available to editors and reviewers. We strongly encourage code deposition in a community repository (e.g. GitHub). See the Nature Portfolio guidelines for submitting code & software for further information.

Data

Policy information about availability of data

All manuscripts must include a data availability statement. This statement should provide the following information, where applicable:

- Accession codes, unique identifiers, or web links for publicly available datasets
- A description of any restrictions on data availability
- For clinical datasets or third party data, please ensure that the statement adheres to our policy

Bulk RNA-Seq data and single nuclei RNA-Seq data that support the findings of this study have been deposited in Gene Expression Omnibus with the primary accession number GSE249414. (To access and review the record GSE249414 while it remains in private status, please use the secure token: wrslqwsevbqbvmd. Once we have a confirmed publishing date, the data will be made publicly available on that day.) All data that supports the findings of this study are available in

Source data file.

Research involving human participants, their data, or biological material

Policy information about studies with human participants or human data. See also policy information about sex, gender (identity/presentation), and sexual orientation and race, ethnicity and racism.

Reporting on sex and gender	N/A
Reporting on race, ethnicity, or other socially relevant groupings	N/A
Population characteristics	N/A
Recruitment	N/A
Ethics oversight	N/A

Note that full information on the approval of the study protocol must also be provided in the manuscript.

Field-specific reporting

Please select the one below that is the best fit for your research. If you are not sure, read the appropriate sections before making your selection.

[x] Life sciences   [ ] Behavioural & social sciences   [ ] Ecological, evolutionary & environmental sciences

For a reference copy of the document with all sections, see nature.com/documents/nr-reporting-summary-flat.pdf

Life sciences study design

All studies must disclose on these points even when the disclosure is negative.

Sample size	Sample size was based on n values needed to evaluate differences between groups in prior studies. No statistical methods were used to predetermine sample size. Statistical analyses used in this study are: one-way or two-way Anova, two-tailed paired or unpaired Student's t test
Data exclusions	No data were excluded from analysis.
Replication	All experiments are represented by multiple biological replicates or independent experiments. The number of replicates per experiment are indicated in the legends. All experiments were conducted using at least two independent experimental materials or cohorts to reproduce similar results.
Randomization	Mice were randomized and evenly distributed to groups for different intervention/treatment based on body weight, phenotype parameters. Whenever possible, group assignments were randomized and the investigators were blinded to allocation during experiments and outcome assessment.
Blinding	Investigators were blinded during the data collection and analysis where possible. This included mice echo, blood pressure, GTT, treadmill exercise stress test, PV-Loop data collection and analysis. The quantification for TMRM, perchlorate assay was performed blindly.

Reporting for specific materials, systems and methods

We require information from authors about some types of materials, experimental systems and methods used in many studies. Here, indicate whether each material, system or method listed is relevant to your study. If you are not sure if a list item applies to your research, read the appropriate section before selecting a response.

Materials & experimental systems

n/a	Involved in the study	
[ ]	[x]	Antibodies
[ ]	[x]	Eukaryotic cell lines
[x]	[ ]	Palaeontology and archaeology
[ ]	[x]	Animals and other organisms
[x]	[ ]	Clinical data
[x]	[ ]	Dual use research of concern
[x]	[ ]	Plants

Methods

n/a	Involved in the study	
[x]	[ ]	ChIP-seq
[x]	[ ]	Flow cytometry
[x]	[ ]	MRI-based neuroimaging

Antibodies

Antibodies used	Primary antibodies included anti-HDAC6 (1:1000; Abcam, ab239362), anti-acetyl-a-tubulin (1:5000; Abcam, ab179484), anti-a-tubulin (1:5000; Abcam, ab7291), tropomyosin (1:1000; Abcam, ab133292), skeletal muscle myosin (1:1000; Santa Cruz, sc-32732), troponin I (1:1000; sc-133117), and anti-GAPDH (1:5000; Abcam, ab181602 and 1:5000; Themo Fisher, MA5-15738). All secondary antibodies were tagged with IRDye (LI-COR Biosciences).
Validation	All these antibodies are commercially available and validated by manufacture and previously done by our or other labs<br>anti-HDAC6 (ab239362), anti-acetyl-a-tubulin (ab179484), anti-a-tubulin (ab7291) and anti-GAPDH (ab181602 and MA5-15738) were validated in our previous publication (DOI: 10.1126/scitranslmed.abl5654)<br>tropomyosin (ab133292) WB application has been validated by vendor (https://www.abcam.com/products/primary-antibodies/tropomyosin-1-alpha--tropomyosin-3-antibody-epr5159-ab133292.html),<br>skeletal muscle myosin (sc-32732), WB application has been validated by SCBT<br>troponin I (sc-133117), WB application has been validated by SCBT

Eukaryotic cell lines

Policy information about cell lines and Sex and Gender in Research

Cell line source(s)	human cardiac fibroblasts (HCFs) were purchased from PromoCell (C-12375) ,iCell Cardiomyocytes2 were ordered from FUJIFILM Cellular Dynamics.
Authentication	None of the cell lines were authenticated in the study
Mycoplasma contamination	All the cell lines were mycoplasma-free
Commonly misidentified lines (See ICLAC register)	No commonly misidentified cell lines were used

Animals and other research organisms

Policy information about studies involving animals; ARRIVE guidelines recommended for reporting animal research, and Sex and Gender in Research

Laboratory animals	Ten-week-old Hdac6 knockout mice were purchased from The Jackson Laboratory (Strain #:029318). Only littermate males from breeding were studied. Ten-week-old male C57BL/6J (Cat. 000664) mice were purchased from The Jackson Laboratory (Bar Harbor, ME). Mice were acclimatied for 1 week before experiments. Mice were housed at 23-25°C with light cycles of 12 h of light beginning at 6:00am and 12 h of dark beginning at 6:00 pm, humidity was 30–70%, and H2O was provided ad libitum.
Wild animals	The study did not involve wild animals.
Reporting on sex	Only male mice used in the study.
Field-collected samples	The study did not involve samples collected from the field.
Ethics oversight	Animal studies complied with regulations of Tenaya Therapeutics (AUP: 2020.005, 2020.007) and the National Institutes of Health.

Note that full information on the approval of the study protocol must also be provided in the manuscript.
